# Topical Tacrolimus and Alcohol-Induced Facial Flushing: A Case Report and Literature Review

**DOI:** 10.7759/cureus.46744

**Published:** 2023-10-09

**Authors:** Volkan Tekmen, Mikayla Cochrane, Joyce Kim, Sylvia Hsu, Adam Rees

**Affiliations:** 1 Dermatology, Sidney Kimmel Medical College at Thomas Jefferson University, Philadelphia, USA; 2 Dermatology, Kaiser Permanente Los Angeles Medical Center, Los Angeles, USA; 3 Dermatology, Temple University Hospital, Philadelphia, USA

**Keywords:** alcohol flushing, facial flushing, calcineurin inhibitor, tacrolimus, case report

## Abstract

Flush reactions can be incited by various factors including inherent mutation, drugs, and diseases. A medication that is commonly used in dermatology but less associated with alcohol-induced facial flushing is topical tacrolimus. We present the case of a 44-year-old man experiencing this phenomenon on a distant, non-application site and a review of cases published in the literature.

## Introduction

Alcohol flush reactions are commonly associated with inherent mutations in aldehyde dehydrogenase, which lead to a buildup of acetaldehyde. Acetaldehyde induces histamine and prostaglandin release, leading to vasodilation [1]. This mutation is particularly prevalent in Asian populations. Additionally, medications such as disulfiram, metronidazole, tinidazole, and trimethoprim/sulfamethoxazole can elicit a similar response, as they inhibit aldehyde dehydrogenase, which leads to acetaldehyde buildup [2,3]. Symptoms may range from erythema and mild warmth to a burning sensation on the face. 

Topical tacrolimus was approved by the FDA in 2002 for the use of atopic dermatitis but is currently prescribed for various inflammatory conditions. It works by binding FK506 binding proteins and inhibiting calcineurin [4]. This leads to a downstream effect of decreasing T-cell activation and inflammatory cytokines, mainly IL-2, IL-4, and IL-5 [4,5].

Flushing with alcohol consumption is an established side effect of calcineurin inhibitors, noted in initial clinical trials as affecting 3.4% and 6.9% of participants using 0.03% and 0.1% formulations, respectively [6]. Despite this relatively high incidence and the potential effect on compliance, this phenomenon is rarely mentioned in patient education. The mechanism by which tacrolimus induces facial flushing is unclear. Here, we present a case of tacrolimus-induced alcohol facial flushing, a review of cases throughout the literature, and a discussion of potential mechanisms and therapeutics. 

## Case presentation

**Figure 1:**
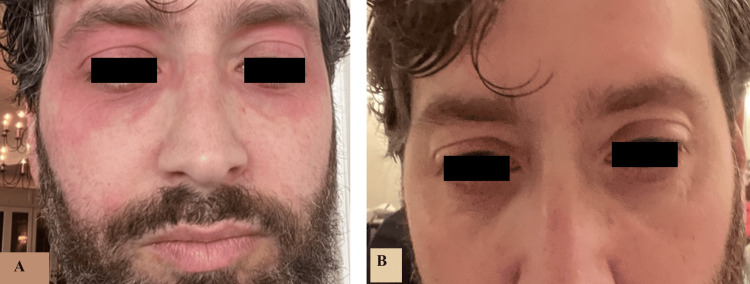
Non-scaly erythematous patches in peri-ocular areas bilaterally: (A) Rash one minute after alcohol consumption and (B) one hour since the flushing onset

A 44-year-old man with a past medical history of plaque psoriasis presented with a complaint of an episodic facial rash that occurred minutes after alcohol consumption. He described the rash as redness and burning around his eyes and ears that lasted up to an hour (Figure 1A). He denied any difficulty breathing during these episodes. The patient reported that his psoriasis was well-controlled using topical medications, which include clobetasol solution and tacrolimus 0.1% ointment. He denied having any known allergies. The patient reported taking four tablets of cetirizine 10 mg upon the onset of flushing, and then the episode resolved within an hour (Figure 1B).

Considering that the patient had no prior history of rash with alcohol intake, it is unlikely that he possesses an inherent mutation in aldehyde dehydrogenase that triggered the flush reactions. Given the lack of history, it is also unlikely that the patient has a condition, such as rosacea that flares with alcohol intake. The patient denied ingesting medications commonly associated with alcohol flushing, including disulfiram, metronidazole, tinidazole, or trimethoprim/sulfamethoxazole. However, upon further investigation, he did report applying tacrolimus to non-facial sites within a few weeks of each flushing episode. As cutaneous flushing with alcohol consumption is an established side effect of tacrolimus and other calcineurin inhibitors, the patient was diagnosed with an alcohol-induced flush reaction secondary to topical tacrolimus use. 

## Discussion

Here, we present a case of alcohol flush reactions due to topical tacrolimus application at a distal site. Several published cases detail alcohol flushing as a side effect of tacrolimus (Table 1). Our case highlights the importance of counseling patients about the possibility of facial flushing outside of application sites, which is not as commonly reported and may be an alarming finding that elicits anxiety and may lead to decreased adherence [7,8]. Additionally, our case demonstrates the typical characteristics that tacrolimus-induced alcohol flush reactions share in the literature.

**Table 1:** Summary of literature reporting tacrolimus-induced alcohol facial flushing

Reference	Number of patients	Medication	Condition	Location (application site, distant site, or both)	Onset after starting tacrolimus	Onset after alcohol ingestion	Time of episode resolution	Resolution of flushing after stopping medication
[6]	6	Topical tacrolimus 0.1% ointment BID	Atopic dermatitis (n=4) Steroid-aggravated rosacea (n=2)	Distant site (face)	5 days	5-15 minutes	30 minutes	4 weeks
[7]	7	Topical tacrolimus (n=5), pimecrolimus (n=2)	Vitiligo	Both	2-4 weeks	5-10 minutes	20-30 minutes	Not reported
[9]	3	Topical tacrolimus	Atopic dermatitis (n=2) and psoriasis (n=1)	Application site (n=2), both (n=1)	Not reported	Immediately	One hour (n=1), not reported (n=2)	Not reported
[10]	1	Tacrolimus 0.1%	Eyelid dermatitis	Application site	Prescribed for 1 week	30 minutes	1-2 hours	Not reported
[11]	1	Tacrolimus 0.1%	Vitiligo	Application site	3 months	20 minutes	30-60 minutes	Not reported
[12]	1	Tacrolimus 0.03%	Pruritic facial papules	Application site	6 months	2 minutes	30 minutes	Not reported

Like our patient, all published case reports describe that flushing episodes are triggered soon after alcohol ingestion, typically within minutes. While one patient experienced flushing as early as two minutes after alcohol consumption, episodes developing after 30 minutes have been reported [3,9]. Even small quantities of alcohol intake can lead to these reactions. Six patients who applied 0.1% tacrolimus ointment developed flushing after consuming only 0.1 L of white wine, while our patient reported flushing after a few sips of alcohol [7]. In line with our case, the literature also suggests that most episodes self-resolve within 20 minutes to an hour of rash onset [7,8,10-13]. Furthermore, while a history of topical calcineurin inhibitor application within the past 1-2 weeks is typically required for flush reactions to occur, they are expected to resolve after a wash-out period of 2-4 weeks [7,8,10-13]. Our patient’s flushing resolved an hour after onset. He reported taking cetirizine 10 mg to treat his episodes, which suggests that antihistamines may be a treatment option to control flushing. However, it could be the case that his flushing episodes would have resolved without treatment, as all published cases in literature self-resolved within an hour.

The mechanism of action and the appropriate treatment approach for alcohol flushing with topical tacrolimus are unclear, and to date, there are no reports of this phenomenon occurring with oral tacrolimus. Currently, there are only proposed mechanisms for tacrolimus flushing. First, it is theorized that tacrolimus causes localized aldehyde dehydrogenase inhibition, leading to the accumulation of acetaldehyde [7]. As acetaldehyde levels elevate in the setting of alcohol ingestion, there may be subsequent vasodilation [7]. Alternatively, a previous study by Ehst et al. investigated prostaglandins as a cause for this flushing phenomenon [14]. The study involved two participants who applied calcineurin inhibitors and control vehicles twice daily for three days on designated areas on the face and forearm. Flushing was noted on the sites of application of tacrolimus and pimecrolimus. Interestingly, the participants had improvement in the flushing reaction with administration of aspirin 325 mg and a worsening with antihistamine use [14]. These findings suggest that prostaglandins, not histamines, may be important downstream mediators for flushing [14].

An additional proposed mechanism for alcohol flushing is that topical tacrolimus induces a capsaicin-like reaction in the skin that leads to an exaggerated response to alcohol. With topical application, tacrolimus causes a burning sensation through the induction of substance P release through extracellular calcium influx. Alcohol may potentiate these effects by further increasing neuropeptide release and causing flushing [15]. Nevertheless, the mechanism of flush reactions remains unclear, and further investigation is required.

## Conclusions

Facial flushing with alcohol intake is a known side effect of calcineurin inhibitors that is not commonly discussed. This report adds to the existing literature by demonstrating a case of tacrolimus-induced flushing at distant, non-application sites with alcohol intake and summarizing the key findings in previous case reports. While no specific treatments have been found, discussing potential side effects of calcineurin inhibitors is important, as flushing reactions can be misinterpreted as a food allergy and lead to unnecessary medical workup. In addition, educating patients on potential time frames of side effects can provide predictability and increase adherence to medication use. 
